# Assessment of exposure to secondhand tobacco smoke in Spain: A scoping review

**DOI:** 10.18332/tid/192118

**Published:** 2024-10-11

**Authors:** Ana Blanco-Ferreiro, Ana Teijeiro, Leonor Varela-Lema, Julia Rey-Brandariz, Cristina Candal-Pedreira, Lucía Martin-Gisbert, Guadalupe García, Iñaki Galán, Esteve Fernández, Nerea Mourino, Mónica Pérez-Ríos

**Affiliations:** 1 Department of Preventive Medicine and Public Health, University of Santiago de Compostela, Santiago de Compostela, Spain; 2 Health Research Institute of Santiago de Compostela, Santiago de Compostela, Spain; 3 Centro de Investigación Biomédica en Red de Epidemiología y Salud Pública, Instituto de Salud Carlos III, Madrid, España; 4 National Centre for Epidemiology, Institute of Health Carlos III, Madrid, Spain; 5 Tobacco Control Unit, Catalan Institute of Oncology, WHO Collaborating Centre for Tobacco Control, L’Hospitalet de Llobregat, Barcelona, Spain; 6 Tobacco Control Research Group, Bellvitge Biomedical Research Institute, L’Hospitalet de Llobregat, Barcelona, Spain; 7 Faculty of Medicine and Health Sciences, University of Barcelona, Barcelona, Spain; 8 Centro de Investigación Biomédica en Red de Enfermedades Respiratorias, Instituto de Salud Carlos III, Madrid, España

**Keywords:** secondhand smoke, questionnaire, Spain, scoping review, observational studies

## Abstract

**INTRODUCTION:**

There is no consensus on the questions that should be included in questionnaires to properly ascertain exposure to secondhand tobacco smoke (SHS). The objective of this study is to analyze the questions included in studies which have assessed SHS exposure in Spain.

**METHODS:**

A scoping review was performed, using PubMed, Embase and Web of Science databases, selecting original articles published in English and Spanish, across the period 2012–2021. We extracted data from each study regarding its design, target population, sample size or geographical scope; we also collected data regarding how studies dealt with exposure to SHS including assessment and intensity of SHS, exposure setting, geographical scope, and the verbatim questions used.

**RESULTS:**

Finally, 75 studies were identified. In the 23 studies carried out in children, verbatim questions were included in 8 studies, and the setting most studied was the home. SHS exposure was assessed during pregnancy and postnatally by 8 studies, the verbatim questions used were described in 2 studies, being exposure ascertained at home and workplace. In the adult population, 14 of 44 studies described the verbatim questions; the setting most studied was the home. Verbatim questions varied among studies.

**CONCLUSIONS:**

Questionnaire-based assessment of SHS exposure is highly heterogeneous, hindering comparability between studies. Therefore, it is necessary to set a standard questionnaire to assess exposure to SHS.

## INTRODUCTION

The harmful health effects of secondhand tobacco smoke (SHS) were first released in the 1960s^1,2^. In 1986, SHS was the main issue of the Surgeon General’s Report entitled ‘The Health Consequences of Involuntary Smoking’^3^, which stated that exposure to SHS was a risk factor for different causes of disease, such as lung cancer, coronary disease in adults, and numerous adverse effects in children, ranging from premature births to sudden infant death syndrome. The Report concluded that there is no safe level of exposure to SHS^4^.

Since then, many epidemiological studies have been performed to obtain detailed information on how many people are exposed to SHS, exposure settings, and the frequency and intensity of such exposure. Studies that use questionnaires to ascertain self-reported SHS exposure, whether as a risk factor or as a variable to be described, are common. Questionnaires allow for obtaining detailed retrospective and updated information at a reasonable cost. That said, however, account must be taken of their limitations, stemming not only from exposure recall bias, but also from individual susceptibility to SHS, or more particularly, from the influence that the different wording of questioning can have on the specific exposure assessed.

Currently, there is no consensus regarding the questions which should be used to assess SHS exposure at the population level^5^. Studies published in the late 1980s concluded that questionnaire-based assessment of SHS exposure underestimated real exposure, since the exposure settings covered were scarce^6^. One study conducted in 2012, oriented to identifying the questionnaires used in Europe to determine SHS exposure, concluded that there had been wide variability in the questions targeted at estimating SHS exposure^7^. Since then, interest in assessing the prevalence of population exposure to SHS has steadily increased, while regulations are being implemented to protect the population from SHS exposure. Therefore, questions addressed to assess SHS exposure are used more frequently in health surveys and epidemiological studies.

Hence, the aim of this study was to identify and describe the questions included in the research studies that have assessed SHS exposure in Spain from 2012 to 2021.

## METHODS

We performed a scoping review in accordance with the PRISMA-ScR (Preferred Reporting Items for Systematic reviews and Meta-Analyses extension for Scoping Reviews) guidelines^8^ and we also followed the recommendations provided by Levac et al.^9^ for advancing scoping review methodology. A search was carried out in PubMed, Embase and Web of Science databases including the following MeSH terms and free terms: tobacco smoke pollution, environmental tobacco smoke, passive smok*, second-hand smoke, secondhand smoke, involuntary smoking, case-control, cohort, prospective, cross-sectional, before–after, Spain and Spanish. The search was limited to original articles published from January 2012 through December 2021. No language restriction was applied, but only studies published in English or Spanish were included. Reviews, letters, comments, clinical cases or case studies, and conference abstracts were excluded. The search strategies used for each database can be found in Supplementary file Table 1.

We selected questionnaire-based research studies that assessed SHS regardless epidemiological design. The target population was classified as children (under 18 years of age) and adults, though studies which simultaneously covered pregnant women and children are shown as a separate category.

Two researchers (ABF and AT) individually reviewed the titles and abstracts of the records identified to select potentially relevant studies. Discrepancies were reviewed by a third reviewer (MPR). The full text of selected articles was then read to ascertain whether they fulfilled the selection criteria. Once studies that met the selection criteria had been identified, the full texts were reviewed by four researchers (ABF, AT, JRB, CCP), with any discrepancies being settled by group discussion and consensus.

Data of interest were recorded on purpose-designed tables, with the following variables being extracted: SHS exposure (designated or not designated as the main study objective); study design (cross-sectional – distinguishing before-after studies, cohort, or case-control); target population (adults, children, pregnant women and children); sample size; questionnaire administration (self-administered – mail or online, face-to-face, telephone, or other); validation of reported exposure (none, cotinine, nicotine, or other); and study scope (local, regional, national – including multicenter studies, or supranational). In each study, we identified the variables related with SHS exposure and extracted data on: the setting covered, both indoors and outdoors (home, workplace-teaching institution, leisure settings, public or private transport, or other); assessment of exposure (presence of smokers, tobacco smell, perception of being ‘exposed’, frequency of exposure, or other). We also collected data on the intensity of exposure, differentiating between the number of cigarettes smoked in their presence, number of smokers, number of places where smoking took place, or other. The verbatim questions on SHS exposure were extracted when available in the selected articles.

## RESULTS

**Figure 1:**
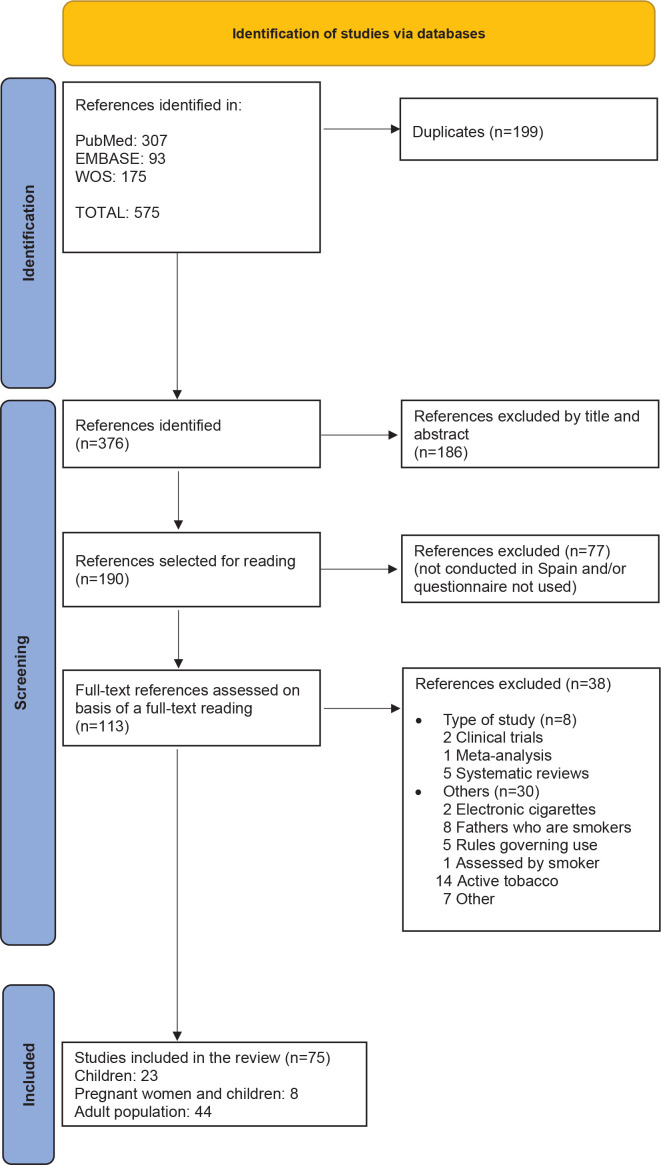
Flowchart of the process of selecting articles included in the study (search in PubMed, Embase and Web of Science, 2012–2021)

The search yielded 575 articles of which 199 were duplicates. After reading the titles and abstracts, 190 articles were read in full text, of which 75 fulfilled the eligibility criteria and were included. Of the 75 articles, 23 assessed exposure to SHS in children, 8 assessed it in pregnant women and children, and 44 assessed it in adults (Figure 1).

### Studies conducted among children

Out of the 23 studies that assessed SHS exposure in children, 6 provided results from the same cohort study (INMA), though assessment of exposure differed among them. In terms of the study design, 14 were cross-sectional studies and 9 were cohort studies. In the cross-sectional studies, the most common geographical scope was regional, accounting for 8 out of 23 studies (Table 1 and Supplementary file Table 2).

**Table 1:** General characteristics of the studies included (N=75)

*Authors*	*Year of publication*	*Year of realization*	*Design*	*Population*	*Settings*
Ortega-García et al.^10^	2012	2009–2010	Cohort	Children	Home-Teaching institution-Leisure
Esplugues et al.^11^	2013	2003–2008			Overall
Fuentes-Leonarte et al.^12^	2015	2003–2008			Home-Teaching institution-Leisure
Mariana Fernández et al.^13^	2015	2000–2002 2005–2006			Home
Aurrekoetxea et al.^14^	2016	2003–2008			Home-Teaching institution-Transport
Robinson et al.^15^	2016	2003–2008			Home-Teaching institution
García-Villarino et al.^16^	2021	2004–2007			Home-Teaching institution-Leisure
Bermudez-Barrezueta et al.^17^	2021	2015–2016			Overall
Maitre et al.^18^	2021	2013–2016			Home
Martín-Pujol et al.^19^	2013	2006	Cross-sectional		Home-Teaching institution-Leisure-Transport
Suárez-López-de-Vergara et al.^20^	2013	2007–2008			Home
Padrón et al.^21^	2014	2008–2009			Home
Padrón et al.^22^	2016	2011–2012			Home
Alicea-Alvarez et al.^23^	2016	2015			Home
Arechavala et al.^24^	2018	2012			Home
López et al.^25^	2018	2016			Home-Teaching institution-Leisure-Transport
Arechavala et al.^26^	2019	2015–2016			Home
Contienente et al.^27^	2019	2015			Home-Teaching institution-Leisure-Transport
Díez-Izquierdo et al.^28^	2019	2017			Home
Lletjós et al.^29^	2020	2016			Home-Teaching institution-Leisure-Transport
			
Henderson et al.^30^	2020	2020			Teaching institution
Continente et al.^31^	2021	2016			Home
Gonzalez-Barcala et al.^32^	2017	2006–2007			Overall
Almendros et al.^33^	2018	2015–2016	Cross-sectional	Pregnant women and children	Home
Hernández-Martínez et al.^34^	2012	2004–2009	Cohort		Home-Workplace/Teaching institution
McBride et al.^35^	2012	2006–2010			Home-Workplace/Teaching institution-Leisure
Casas et al.^36^	2013	2004–2006			Home
Ribot et al.^37^	2014	2005–2008			Home-Workplace/Teaching institution
Hernández-Martínez et al.^38^	2017	2005–2009			Home-Workplace/Teaching institution
Roigé-Castellví et al.^39^	2020	2005–2014			Home-Workplace/Teaching institution
Iniguez et al.^40^	2016	2003–2008			Home-Workplace-Leisure
Ruano-Ravina et al.^41^	2014	2011–2013	Case-control	Adults	Home
Torres-Durán et al.^42^	2014	2011–2013			Home
Almirall et al.^43^	2014	1999–2000			Home
Torres-Durán et al.^44^	2015	2011–2013			Home
Torres-Durán et al.^45^	2017	2011–2016			Home-Workplace/Teaching institution
González-Romero et al.^46^	2018	2015			Home
Molina-Montes et al.^47^	2020	2007			Overall
Torres-Durán et al.^48^	2021	2011–2019			Home
Torres-Durán et al.^49^	2015	2011–2013			Overall
Sunyer et al.^50^	2012	2004–2008	Cohort		Home-Workplace-Leisure
Larrañaga et al.^51^	2013	2003–2008			Home-Workplace/Teaching institution-Leisure
Ortega-García et al.^52^	2016	2008–2013			Home
Lidón-Moyano et al.^53^	2017	2013–2014			Home-Workplace/Teaching institution-Leisure-Transport
Pérez-de-Arcelus et al.^54^	2017	2011			Home-Workplace/Teaching institution
Román-Gálvez et al.^55^	2018	2013–2015			Home
Flexeder et al.^56^	2019	1990–1994 1998–2001			Overall
Olivieri et al.^57^	2019	1998–2003 2010–2014			Home-Workplace/Teaching institution
Íñiguez et al.^58^	2012	2004–2006			Home-Workplace/Teaching institution
Ruano-Ravina et al.^59^	2020	2018–2019			Overall
Villaverde-Royo et al.^60^	2012	2009–2011	Cross-sectional		Overall
Clemente-Jiménez et al.^61^	2012	2008			Home-Workplace/Teaching institution-Leisure-Transport
Martínez Sánchez et al.^62^	2012	2004–2005			Home-Leisure
Ortega-García et al.^63^	2012	2008			Home-Workplace/Teaching institution-
					Leisure-Transport
Jimenez-Muro et al.^64^	2012	2009–2010			Home-Workplace/Teaching institution
Aurrekoetxea et al.^65^	2013	2004–2008			Home-Workplace/Teaching institution-Leisure
Mateos-Vílchez et al.^66^	2014	2007–2012			Home-Workplace/Teaching institution-Leisure
Sureda et al.^67^	2014	2004–2005 2011–2012			Home-Workplace/Teaching institutionLeisure-Transport
Aurrekoetxea et al.^68^	2014	2004–2008			Home-Workplace/Teaching institution-Leisure
Pérez-Ríos et al.^69^	2014	2005–2011			Home-Workplace/Teaching institution-Leisure
Galán et al.^70^	2014	2010			Leisure
Sureda et al.^71^	2015	2011–2012			Home-Workplace/Teaching institution-Leisure-Transport
Ballbè et al.^72^	2015	2010–2011			Home-Workplace/Teaching institution
Ballbè et al.^73^	2015	2011–2012			Home-Workplace/Teaching institution-Transport
Fernández et al.^74^	2017	2006–2011			Home-Workplace/Teaching institution-Leisure-Transport
Martínez et al.^75^	2017	2014–2015			Teaching institution
Viñolas et al.^76^	2017	2007–2012			Home-Workplace/Teaching institution
Martínez Sánchez et al.^77^	2018	2011–2012			Home
Sureda et al.^78^	2018	2016			Leisure
Fu et al.^79^	2018	2013			Leisure
Míguez et al.^80^	2020	2012–2015			Home
Lidón-Moyano et al.^81^	2021	2013–2014			Home-Workplace/Teaching institution-Leisure-Transport
Rebollar-Álvarez et al.^82^	2021	2020			Home
Henderson et al.^83^	2021	2017–2018			Teaching institution-Leisure-Transport
Nogueira et al.^84^	2021	2017–2018			Workplace/Teaching institutionLeisure-Transport

**Figure 2:**
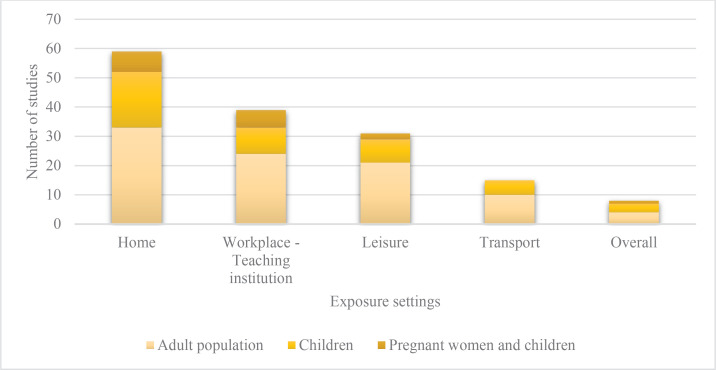
Secondhand smoke exposure settings assessed in the studies (N=75)

In 17 studies, SHS exposure-related aspects was the main objective (Table 2). The settings most commonly assessed were the home (19 of 23), followed by teaching institution (10 of 23) (Figure 2). Nine of the 23 articles included determined objective exposure markers, mainly cotinine. Verbatim questions were included in 8 of the 23 articles, including different questions to ascertain SHS exposure for the same setting (Table 1 and Supplementary file Table 2). Studies included different SHS exposure indicators, with the presence of smokers being the most commonly used to assess SHS exposure in the home setting (8 of 19), workplace-teaching institution (2 of 9) and transport (3 of 5); and the perception of being exposed to assess SHS exposure in leisure (3 of 8). The intensity of SHS exposure was assessed (3 of 23) by ‘number of hours exposed’ and ‘number of smokers’ (Table 3).

**Table 2:** Characteristics of included studies related to the assessment of exposure to secondhand tobacco smoke. The number of studies assessing each characteristic by type of population is shown (N=75)

*Characteristics*	*Children (N=23)*	*Pregnant women and children (N=8)*	*Adults (N=44)*
**Secondhand smoke main objective**			
Yes	17	5	26
No	6	3	18
**Sample size**			
<500	7	5	13
500–1000	1	1	8
>1000	15	2	23
**Validation self-reported exposure**			
Cotinine	6	2	13
Nicotine	2	0	4
Other	0	1^a^	3^a^
No	15	5	27
**Geographical scope**			
Local	6	6	15
Regional	8	1	20
National	7	0	4
Supra-national	2	1	5
**Number of settings**			
Global	3	1	5
1	8	1	15
2	3	5	6
3	2	1	7
>3	7	0	11
**Verbatim questions**			
Yes	8	2	14
No	15	6	30

a Other: benzene, particulate matter (PM2.5).

**Table 3:** Indicators of exposure to secondhand tobacco smoke assessed in the included studies and number of studies analyzing them by exposure setting (N=75)

*Exposure indicators*	*Home*			*Workplace and Teaching institution*				*Leisure*		*Transport*		
	*Children (N=19)*	*Pregnant women and children (N=7)*	*Adults (N=33)*	*Children (N=9)*	*Pregnant women and children (N=6)*	*Adults (N=24)*	*Children (N=8)*	*Pregnant women and children (N=2)*	*Adults (N=21)*	*Children (N=5)*	*Pregnant women and children (N=0)*	*Adults (N=10)*
Presence of smokers	13	0	18	2	0	8	1	0	3	3	0	4
Tobacco smell	1	2	0	1	2	0	1	0	2	0	0	0
Perception of being ‘exposed’	1	0	4	1	0	7	3	0	2	1	0	0
Smoking area (indoor vs outdoor)	4	0	3	1	0	2	0	0	4	0	0	1
Number of times per week	2	0	3	1	0	0	2	0	4	0	0	0
Not assessed	2	4	9	2	3	1	1	1	7	0	0	5
Assess more than one	5	0	4	1	0	1	1	0	2	0	0	0

The period for which recall was elicited was variable: at home, parents or guardians were asked about generic exposures or exposures in the previous two weeks; and in teaching institution, parents or guardians were asked about exposure in the preceding week (Table 1 and Supplementary file Table 2).

### Studies on pregnant women and children

The search identified 8 cohort studies that assessed exposure to SHS in pregnant women and their children, with 3 of these providing results on the DEFENSAS study (Table 1 and Supplementary file Table 2). The main stated objective of 5 of the 8 articles was to study SHS exposure (Table 2). The SHS exposure was most commonly assessed in the home setting (7 of 8), followed by the workplace and teaching institution (5 of 8) (Figure 2). SHS exposure was assessed during pregnancy (prenatal exposure) in 7 studies, and postnatally in one. Exposure to SHS was established by reference to ‘tobacco smell’ in 2 out of the 8 studies that assessed exposure to SHS at home and in 2 out of the 5 studies that assessed SHS at the workplace. Intensity of exposure was assessed by ‘number of hours’ by one study (Table 3).

### Studies on the adult population

Of the 44 studies that assessed SHS exposure in adults, 5 were based on the INMA cohort study. Regarding the study design, 25 out of the 44 studies in adults were cross-sectional, 9 were cohort studies, and 10 were case-control studies (Table 1 and Supplementary file Table 2). Of the 44 studies, 26 stated that their main objective was to study SHS exposure. The articles referred mainly to studies conducted at a local (15 of 44) and regional level (20 of 44). Objective exposure markers were ascertained in 17 of 44 studies measuring cotinine or nicotine (Table 2). The most commonly studied setting was home (33 of 44), followed by the workplace and teaching institution (24 of 44), and leisure (20 of 44) (Figure 2). Five studies assessed exposure in workplaces, as well as asking subjects about exposure in outdoor settings (Table 1 and Supplementary file Table 2). Assessment of SHS in the home focused mainly on recall of exposure during the previous week. The time window in the workplace/place of study was more variable and ranged from the preceding week to more than 1 year or an indefinite period. Exposure in the home was mainly achieved by asking about the presence of smokers (18 of 33). Intensity of SHS exposure was assessed on the basis of number of times per week exposed to SHS in 9 of 44 studies (Table 3).

## DISCUSSION

The results of our study show a wide variability in how exposure to SHS is assessed in research studies conducted in Spain. Exposure to SHS was more frequently assessed in the home setting, followed by the workplace and teaching institution, leisure, and, less frequently, transport. Despite being considered of utmost importance to assess all possible settings^85^, assessment of SHS exposure in other settings, such as outdoor areas, is anecdotal. The inclusion of the verbatim questions used to elicit exposure is unusual. Our analysis reveals that there is a wide variability in the way SHS exposure is assessed^86,87^, i.e. the questions are investigation group- or study-dependent.

A previous study concluded that questionnaires underestimated the real prevalence of exposure. The study indicated that this may be because the assessment of exposure is limited to two places: the home and the workplace^88^. Our study indicates that in the most recent studies, the number of settings in which exposure is assessed by questionnaires has increased, and that it is now more common to include other settings, such as leisure settings. Nonetheless, assessment of exposure in settings such as transport or in outdoor areas is still very infrequent. Since there is no clearly safe SHS exposure threshold, assessing exposure in all settings where exposure may take place seems essential. This would also help address another relevant aspect, namely, that of defining who should consider himself or herself exposed to SHS. An exposed person should be anyone who reports exposure, regardless of its setting, duration or intensity. In light of this, prudence is called for studies to assess the duration and/or intensity of exposure; this could avoid the over-reporting associated with accidental or anecdotal exposures. Assessment of accidental exposures to low SHS concentrations could be influenced by the susceptibility of the person who reports exposure, thereby giving rise to differential reporting bias.

It should also be noted that although there are studies that assess SHS worldwide, such as the Global Adult Tobacco Survey (GATS), the questions included in these studies supported by organizations such as WHO, are not replicated nor is the definition of exposure in studies at the national or local level. It is imperative to advance in the standardization of questions aimed at determining exposure and to reach a global agreement to define who is exposed to SHS.

Another aspect to be highlighted is the researchers’ tendency to systematically omit the questions that they use to assess exposure. This is an important limitation, since if questions are not included, it would be impossible to critically assess the results of studies and contextualize them, given the current lack of standardized set of questions. If question omission is of the researcher’s own volition or caused by journals’ editorial review processes (which tend to put short explanations before detail), then it becomes a more complicated topic that cannot be addressed. In those studies that do provide the questionnaires, there is a surprisingly wide variation in the formulation of questions.

An additional point to highlight is the objective assessment of exposure to SHS. In 25 studies, exposure to SHS was assessed with biomarkers, principally cotinine. Nevertheless, determination of cotinine was performed sometimes to differentiate smokers from non-smokers, not to assess different levels of exposure to SHS. While some studies show agreement between both measures^65,68^, others display discrepancies^58^. It should be taken into account that such validation in studies at a population level has no logical basis because the period of time covered by the subject’s recollection of when exposure occurred as shown in the questionnaire and the exposure time window covered by the biomarker are usually different. Exposure biomarkers such as cotinine are detectable in human biological samples with a high degree of precision and allow for approximation of the exposure dose, and despite their half-life, are relatively constant throughout the day^89^. However, conducting a study to determine cotinine in a representative population sample would have some limitations. The first – and extremely important – limitation resides in the difficulty and cost of conducting such a study. Furthermore, there are discrepancies regarding which cut-off point to apply, something that, for instance, varies considerably in children^90^, being also inadequate to assess past exposures. Furthermore, the advances in the techniques of analysis used to quantify cotinine might also amount to a limitation, since current techniques are very sensitive, and therefore, detect very low cotinine concentrations which would be linked to non-meaningful exposures. It should also be stressed here that neither cotinine nor any other biomarker can provide information regarding the place of exposure if no information about the place of exposure is also collected. For this purpose, questionnaire-based data-collection is indispensable.

Nonetheless, assessing exposure to SHS at a population level is complicated because there is great variability in terms of the settings where people are exposed, source of exposure, the SHS concentration, the duration of exposure, the characteristics of the person concerned, and his/her history of exposure. Moreover, the main aim of the study can have a marked influence on the questions to be included. Hence, studies targeted at assessing the impact of SHS exposure on health should prioritize history of exposure over exposure settings, whereas studies targeted at estimating the prevalence of exposure should prioritize exposure settings over history of exposure. Failure to include the correct questions may trigger misclassification of SHS exposure, and lead, among other things, to the poor performance of early interventions based on primary prevention, incorrect evaluation of smoking control policies, or inaccurate estimation of the impact associated with exposure. While identification of the various settings in which exposure can occur, assessment of exposure indicators, measurement of the intensity of exposure, and precise characterization of the history of exposure, would allow for a correct characterization of the exposure and are all essential for assessing SHS exposure accurately.

Giving the difficulty of generating a single set of questions to assess exposure to SHS, it seems important to draw a distinction, *a priori*, between a study targeted at estimating prevalence of exposure and one targeted at assessing the relationship between SHS exposure and a health outcome. In either case, pinpointing those characteristics specific to the survey respondent which may determine response, such as smoker status or tolerance to smoking, is important to prevent reporting biases. Several initiatives such as the Global Youth Tobacco Survey (GYTS)^91,92^, recommend that exposure to SHS should be assessed with a breakdown of such exposure in closed spaces. Breaking down exposure in the home, workplace-teaching institution, leisure or transport is indispensable, as is having an indicator of the duration or intensity of such exposure. These last two aspects are crucial when conducting studies targeted at assessing the relationship between exposure and a health outcome, along with an accurate and detailed history of exposure.

Having questions that allow a breakdown of where exposure occurs is also important for the evaluation of the implementation of new tobacco control policies. In Spain, where a modification of the tobacco control law is expected soon, scientific societies are calling for further progress in protecting the population from exposure to SHS by expanding smoke-free spaces. These spaces include hospitality terraces, sports facilities, university campuses, transport stops, swimming pools, beaches, and natural areas.

### Limitations

This study has some limitations. One limitation might be not having included studies prior to 2012, but this information was included in two previous reviews, one focused on Europe^7^ and the other on informal sources on SHS exposure in Spain^86^. This review focuses on the questions used to assess SHS exposure in Spain. However, there does not seem to be any justification to assume that the variability in the questions used in Spain is not common in other countries. A quantitative synthesis of the results was not possible in this review because of the nature of the results extracted. In addition, as the aim of this review was to analyze the questions used to estimate exposure to SHS, an analysis of the individual potential risks of bias of each of the included studies was not included. This review’s main advantage lies in the fact that it includes studies not only assessing SHS exposure as the main dependent or independent variable, but also studies including SHS exposure as independent secondary variable or variable of adjustment. Furthermore, we included studies conducted on both adults and children, creating a separate category for those that simultaneously assessed pregnant women and children.

## CONCLUSIONS

We would like to highlight the importance of obtaining SHS exposure in a more standardized manner through questions. Having a set of recommendations and standardized questions for assessing SHS exposure, would allow for comparable data to be obtained. Furthermore, it would be important to generate specific groups of questions for each epidemiological design or study objective. Currently, it is not known whether differences in exposure are due to real differences or to data-collection differences. In fact, changes in prevalence of exposure to SHS in a period of time could be just an artifact if the way to measure exposure is constantly changing. Lastly, it should be borne in mind that any questionnaire-based measurement process will inevitably be associated with an error which will have to be accepted. In view of these results, a standardized questionnaire for obtaining self-reported exposure to SHS from individuals should be established and it should be used by researchers to assess an individual SHS exposure. This questionnaire should be made up of sets of questions that would enable SHS exposure to be measured in a harmonized manner. Meanwhile, the questions used to assess exposure should be included in scientific articles. This would allow readers to know which settings are assessed, what time frame the exposure refers to or whether other aspects such as intensity or frequency of exposure are assessed. Readers will then be able to make a proper judgement on whether characterization of SHS exposure is relatively more or less appropriate.

## Supplementary Material



## Data Availability

The data supporting this research are available from the authors on reasonable request.
