# Supplementary material for: Assessment of exposure to secondhand tobacco smoke in Spain: A scoping review
Source: Tob Induc Dis. 2024 Oct 11;22:10.18332/tid/192118. doi: 10.18332/tid/192118 (PMC11468508; doi:10.18332/tid/192118)
Supplement: Supplementary file 1 [file TID-22-165-s1.pdf]

Supplementary material

Table 1. Search strategies used in databases.

Search strategy used in PubMed

**1**	Tobacco smoke pollution[MeSH Terms]	14,932
**2**	Environmental tobacco smoke	17,501
3	Passive smok*	6,983
**4**	Second-hand smoke	16,648
**5**	secondhand smoke	17,572
**6**	involuntary smoking	16,148
**7**	1 or 2 or 3 or 4 or 5 or 6	30,618
**8**	case-control	395,482
**9**	cohort	1,072,990
**10**	prospective	1,117,759
**11**	cross-sectional	716,271
**12**	before-after	7,944
**13**	8 or 9 or 10 or 11 or 12	2,861,095
**14**	Spain	738,117
**15**	Spanish	498,478
**16**	14 or 15	1,134,867
**17**	("2012/01/01" [Date - Publication] : "2021/12/31" [Date - Publication]	11,934,287
**18**	7 AND 13 AND 16 AND 17	307

Search strategy used in EMBASE

**1**	tobacco smoke pollution or environmental tobacco smoke or passive smok* or secondhand smoke or second hand smoke or involuntary smoke).mp. [mp=title, abstract, heading word, drug trade name, original title, device manufacturer, drug manufacturer, device trade name, keyword heading word, floating subheading word, candidate term word]	23,159
**2**	(case control or cohort or prospective or cross sectional or before after).mp. [mp=title, abstract, heading word, drug trade name, original title, device manufacturer, drug manufacturer, device trade name, keyword heading word, floating subheading word, candidate term word]	3,879,063
**3**	(Spain or Spanish).mp. [mp=title, abstract, heading word, drug trade name, original title, device manufacturer, drug manufacturer, device trade name, keyword heading word, floating subheading word, candidate term word]	215,231
**4**	1 and 2 and 3	165
**5**	limit 4 to yr="2012 - 2021"	93

Search strategy used in Web of Science

**1**	TS=(tobacco smoke pollution)	22,668
**2**	TS=(environmental tobacco smoke)	26,741
3	TS=(passive smok*)	15,983

**4**	TS=(second-hand smoke)	3,056
**5**	TS=(secondhand smoke)	6,521
**6**	TS=(involuntary smoking)	824
**7**	#1 or #2 or #3 or #4 or #5 or #6	49,342
**8**	TS=(case-control)	463,455
**9**	TS=(cohort)	1,528878
**10**	TS=(prospective)	1,346,489
**11**	TS=(cross-sectional)	1,430,432
**12**	TS=(before-after)	15,009
**13**	#8 or #9 or #10 or #11 or #12	4,221,233
**14**	TS=(Spain)	378,810
**15**	TS=(Spanish)	293,507
**16**	#14 or #15	594,718
**17**	DOP=(2012-01-01/2021-12-31)	35,477,060
**18**	#7 AND #13 AND #16 AND #17	175

Table 2. Studies included in the review.

Author	Year of publication
Ortega-García et al[1]	2012
Esplugues et al[2]	2013
Fuentes-Leonarte et al[3]	2015
Mariana Fernández et al[4]	2015
Aurrekoetxea et al[5]	2016
Robinson et al[6]	2016
García-Villarino et al[7]	2021
Bermudez-Barrezueta et al[8]	2021
Maitre L et al[9]	2021
Martín-Pujol et al[10]	2013
Suárez-López-de-Vergara et al[11]	2013
Padrón et al[12]	2014
Padrón et al[13]	2016
Alicea-Alvarez et al [14]	2016
Arechavala et al[15]	2018
López et al[16]	2018
Arechavala et al[17]	2019
Contienente et al[18]	2019
Díez-Izquierdo et al[19]	2019
Lletjós et al[20]	2020
Henderson et al[21]	2020
Continente et al[22]	2021
Gonzalez -Barcala et al[23]	2017
Almendros RR et al[24]	2018
Hernández-Martínez et al[25]	2012
McBride et al[26]	2012
Casas et al[27]	2013
Ribot et al[28]	2014
Hernández-Martínez et al[29]	2017
Roigé-Castellví et al[30]	2020
Iniguez C et al[31]	2016

Ruano-Ravina et al[32]	2014
Torres-Durán et al[33]	2014
Almirall et al[34]	2014
Torres-Durán et al[35]	2015
Torres-Durán et al[36]	2017
González-Romero et al[37]	2018
Molina-Montes et al[38]	2020
Torres-Durán et al[39]	2021
Torres-Durán et al[40]	2015
Sunyer et al[41]	2012
Larrañaga et al[42]	2013
Ortega-García et al[43]	2016
Lidón-Moyano et al[44]	2017
Pérez-de-Arcelus et al[45]	2017
Román-Gálvez et al[46]	2018
Flexeder et al[47]	2019
Olivieri et al[48]	2019
Íñiguez et al[49]	2012
Ruano-Ravina et al[50]	2020
Villaverde-Royo et al[51]	2012
Clemente-Jiménez et al[52]	2012
Martínez Sánchez et al[53]	2012
Ortega-García et al[54]	2012
Jimenez-Muro et al[55]	2012
Aurrekoetxea et al[56]	2013
Mateos-Vílchez et al[57]	2014
Sureda et al[58]	2014
Aurrekoetxea et al[59]	2014
Pérez-Ríos et al[60]	2014
Galán et al[61]	2014
Sureda et al[62]	2015
Ballbè et al[63]	2015
Ballbè et al[64]	2015
Fernández et al[65]	2017
Martínez et al[66]	2017

Viñolas et al[67]	2017
Martínez Sánchez et al[68]	2018
Sureda et al[69]	2018
Fu et al[70]	2018
Míguez et al[71]	2020
Lidón-Moyano et al[72]	2021
Rebollar-Álvarez et al[73]	2021
Henderson et al[75]	2021
Nogueira et al[74]	2021

Table 3. Question wording for secondhand smoke exposure questions

**CHILDREN**
**Home**
"¿Conviven fumadores con el niño?" [4]<br>["Is your child exposed to secondhand smoke from household members who smoke?"]
"¿Qué situación describe mejor las "normas" para fumar dentro de su casa?" "Durante las 2 últimas semanas, ¿tu hijo/a ha estado expuesto pasivamente al tabaco en tu casa? ¿Y en otro lugar que no sea tu casa?"[19]<br>**["Which situation best describes the "rules" for smoking inside your home?"** "During the last 2 weeks, has your child been exposed to secondhand smoke in your home? And somewhere else than your home?"]
"¿Cuántas personas que viven contigo en casa fuman habitualmente en casa (sin incluir fumar en el balcón o terraza)?" "En la escuela, ¿hueles humo de tabaco en alguno de los siguientes lugares?" "¿Suele pasar tiempo en lugares cerrados, ni en casa ni en la escuela, donde alguien fuma (tan cerca de usted que puede oler el humo)?" [10]<br>["How many household members usually smoke at home (not including smoking on the balcony or terrace)?" "At school, do you smell tobacco smoke in any of the following places?" "Do you often spend time in enclosed places, neither at home nor at school, where someone smokes (so close to you that you can even smell the tobacco smoke)?"]
"¿Fuma dentro de casa?" "¿Fuma en lugares cerrados como terrazas, balcones, galerías, jardines...?" "Además de los familiares, ¿alguien tiene fumado dentro de la casa en la última semana?" y "¿y en lugares al aire libre como terrazas y balcones?" [17]<br>["Do you smoke inside the home?" "Do you smoke in enclosed places such as terraces, balconies, galleries, gardens...?" "Apart from the household members, has anyone smoked indoors in the last week?" and "What about outdoor places such as terraces and balconies?"]
"¿Fuma algún conviviente con el niño? ¿Cuántos cigarrillos fuma al día?" [11]<br>["Does any household member smoke in the presence of the child?? How many cigarettes do they smoke per day?"]

“¿Fumó usted durante el embarazo? Si fumo diariamente, ¿Cuántos cigarrillos fumó?” “¿La madre fuma? Si la madre fuma diariamente, ¿Cuántos cigarrillos fuma? ¿El padre fuma? Si el padre fuma diariamente, ¿Cuántos cigarrillos fuma?” [8] ["Did you smoke during pregnancy? If daily, how many cigarettes did you smoke?" "Does the mother smoke? If the mother smokes daily, how many cigarettes does she smoke? Does the father smoke? If the father smokes daily, how many cigarettes does he smoke?"]
**Teaching institution**
“¿Durante la última semana, ¿alguien ha fumado en la puerta de entrada/salida en su presencia?” [16] ["During the last week, has anyone smoked at the entrance/exit door in your presence?"]
“Durante la última semana, ¿alguien ha fumado en la puerta de entrada/salida de la guardería o de la escuela del menor en su presencia?” [20] ["During the last week, has anyone smoked at the entrance/exit door of the child's daycare centre or school in his/her presence?”]
**<u>PREGNANT WOMEN AND CHILDREN</u>**
**Home**
“¿Suele oler el humo del tabaco en casa?” “¿Suele oler humo de tabaco en su lugar de trabajo?” [25,29] ["Do you usually smell tobacco smoke at home?" "Do you usually smell tobacco smoke at your workplace?"]
**<u>ADULT POPULATION</u>**
**Home**
“Durante la semana pasada, ¿cuántos cigarrillos se han fumado al día en su presencia en su casa?” (entre semana o día laborable / fin de semana) “¿Cuánto tiempo de «promedio» acostumbra a estar en ambientes con humo de tabaco fuera de casa y del trabajo?” (entre semana o día laborable / fin de semana) [53] ["During the last week, how many cigarettes have been smoked per day at home in your presence?" (during weekday or weekend) "How much time “on average” do you usually spend in smoke-filled environments outside your home and workplace?" (during weekday or weekend)]

“Actualmente, ¿cuántas personas fuman habitualmente dentro de su hogar por día?” “Durante la última semana, ¿cuántos cigarrillos (por día) se han fumado en su presencia dentro de su hogar?” [72]<br>["Currently, how many people usually smoke inside your home on a daily basis?" "During the last week, how many cigarettes (per day) have been smoked inside your home in your presence?"]
“¿Actualmente vive con uno o más fumadores en casa?” [34]<br>["Do you currently live with one or more smokers at home?"]
“La semana pasada (desde el lunes al domingo), ¿con qué frecuencia piensa que ha estado expuesto al humo de tabaco en [casa/trabajo/ocio]?” [60]<br>["During the last week (from Monday to Sunday), how often do you think you have been exposed to tobacco smoke in [home/workplace/leisure place]?"]
“¿Ha vivido o trabajado con un fumador en la misma habitación durante más de un año?” [45]<br>[“Have you lived or worked with a smoker in the same room for more than a year?"]
“Actualmente, ¿cuántas personas por día fuman habitualmente dentro de su hogar?” y” Durante la última semana, ¿cuántos cigarrillos (por día) se han fumado en su presencia dentro de su hogar?” [58]<br>["Currently, how many people per day usually smoke inside your home?" and" During the last week, how many cigarettes (per day) have been smoked inside your home in your presence?"]
“Durante la última semana, ¿cuántas personas por día fumaron habitualmente dentro de su hogar?” “Durante la última semana, ¿cuántos cigarrillos (por día) se han fumado en su presencia en la habitación donde se instalaron los dispositivos?” “Durante la última semana, ¿cuántas horas (por día) ha estado expuesto en la habitación donde se instalaron los dispositivos?” [68]<br>["During the last week, how many people per day usually smoked inside your home?" "During the last week, how many cigarettes (per day) were smoked in the room where the devices were installed in your presence?" "During the last week, how many hours (per day) were you exposed to secondhand smoke in the room where the devices were installed?"]

“Actualmente, ¿cuántas personas suelen fumar dentro de su casa por día?” y “Durante la última semana, ¿cuántos cigarrillos por día se han fumado en su presencia dentro de su casa?” [44]<br>["Currently, how many people usually smoke inside your home on a daily basis?" and "During the last week, how many cigarettes per day have been smoked inside your home in your presence?"]
"¿Cómo describiría el humo de segunda mano en este lugar de hostelería? Intensidad de exposición alta, media, baja y muy baja" "¿Qué puntuación de 0 a 10 le daría a este lugar de hostelería con respecto a la cantidad de humo de tabaco, teniendo en cuenta que 0 sería contaminación mínima y 10 contaminación máxima?" [61]<br>[“How would you describe the second-hand smoke in this hospitality venue? High, medium, low, and very low intensity of exposure” “What score from 0 to 10 would you give this hospitality venue regarding the amount of tobacco smoke, bearing in mind that 0 would be minimum contamination and 10 maximum contamination?”]
**Workplace - Teaching institution**
“La semana pasada (desde el lunes al domingo), ¿con qué frecuencia piensa que ha estado expuesto al humo de tabaco en [casa/trabajo/ocio]?” [60]<br>["During the last week (from Monday to Sunday), how often do you think you have been exposed to tobacco smoke at [home/workplace/leisure place]?"]
“¿Ha vivido o trabajado con un fumador en la misma habitación durante más de un año?” [45]<br>["Have you lived or worked with a smoker in the same room for more than a year?"]
“¿Alguien fuma cerca de usted en el trabajo?” y “¿Cuántas horas al día cree que está expuesto al humo del tabaco en su lugar de educación?” [58]<br>["Does anyone smoke near you at workplace?" and "How many hours a day do you think you are exposed to tobacco smoke at your teaching institution?"]
“¿La gente fuma regularmente en la sala donde trabaja?”” ¿Cuántas horas al día está expuesto al humo de tabaco de otras personas?” [48]<br>["Do people usually smoke in your work room?" "How many hours a day are you exposed to secondhand tobacco smoke?"]

"¿Alguien fuma cerca de usted en el trabajo?" "¿Cuántas horas al día cree que está expuesto al humo del tabaco en su lugar de educación?" [65]<br>["Does anyone smoke near you at workplace?" "How many hours a day do you think you are exposed to tobacco smoke at your educational institution?"]
**Transport**
"Durante la última semana, ¿estuvo usted en un vehículo de transporte público mientras alguien fumaba?" y "Durante la última semana, ¿estuvo usted en un vehículo de transporte privado mientras alguien fumaba?" [58]<br>["During the last week, were you in a public vehicle where someone was smoking?" and "During the last week, were you in a private vehicle where someone was smoking?"]
**Leisure**
"La semana pasada (desde el lunes al domingo), ¿con qué frecuencia piensa que ha estado expuesto al humo de tabaco en [casa/trabajo/ocio]?" [60]<br>["During the last week (from Monday to Sunday), how often do you think you have been exposed to tobacco smoke at [home/workplace/leisure place]?"]
"En los últimos 6 meses, ¿hubo personas fumando cigarrillos regulares la última vez que visitó los siguientes sitios?" (terrazas de restaurantes y bares, paradas de transporte público, áreas al aire libre de hospitales, áreas al aire libre de escuelas, parques, parques infantiles, estadios y playas) [74,75]<br>["In the last 6 months, were there people smoking regular tobacco cigarettes the last time you visited the following places" (restaurant and bar terraces, bus stops, outdoor areas of hospitals, outdoor areas of schools, parks, playgrounds, stadiums and beaches)?"]
**All over**
"¿Cuánto tiempo ha pasado en algún lugar con humo de tabaco que no sea su casa o su trabajo?" [58]<br>["How long have you spent somewhere, other than your home or workplace, exposed to secondhand tobacco smoke?"]
"¿Ha estado expuesto regularmente al humo del tabaco en los últimos 12 meses?" [47]<br>["Have you been exposed to tobacco smoke on a regular basis in the last 12 months?"]

REFERENCES

1. Ortega-Garcia JA, Lopez-Fernandez MT, Llano R, et al. Smoking prevention and cessation programme in cystic fibrosis: integrating an environmental health approach. *J Cyst Fibros*. Jan 2012;11(1):34-9. doi:10.1016/j.jcf.2011.09.005
2. Esplugues A, Estarlich M, Sunyer J, et al. Prenatal exposure to cooking gas and respiratory health in infants is modified by tobacco smoke exposure and diet in the INMA birth cohort study. *Environ Health*. Dec 1 2013;12(1):100. doi:10.1186/1476-069X-12-100
3. Fuentes-Leonarte V, Estarlich M, Ballester F, et al. Pre- and postnatal exposure to tobacco smoke and respiratory outcomes during the first year. *Indoor Air*. Feb 2015;25(1):4-12. doi:10.1111/ina.12128
4. Fernandez MF, Artacho-Cordon F, Freire C, et al. Trends in children's exposure to second-hand smoke in the INMA-Granada cohort: an evaluation of the Spanish anti-smoking law. *Environ Res*. Apr 2015;138:461-8. doi:10.1016/j.envres.2015.03.002
5. Aurrekoetxea JJ, Murcia M, Rebagliato M, et al. Second-hand smoke exposure in 4-year-old children in Spain: Sources, associated factors and urinary cotinine. *Environ Res*. Feb 2016;145:116-125. doi:10.1016/j.envres.2015.11.028
6. Robinson O, Martinez D, Aurrekoetxea JJ, et al. The association between passive and active tobacco smoke exposure and child weight status among Spanish children. *Obesity (Silver Spring)*. Aug 2016;24(8):1767-77. doi:10.1002/oby.21558
7. Garcia-Villarino M, Fernandez-Iglesias R, Riano-Galan I, et al. Prenatal Exposure to Cigarette Smoke and Anogenital Distance at 4 Years in the INMA-Asturias Cohort. *Int J Environ Res Public Health*. Apr 29 2021;18(9)doi:10.3390/ijerph18094774
8. Bermudez Barrezueta L, Minambres Rodriguez M, Palomares Cardador M, et al. Effect of prenatal and postnatal exposure to tobacco in the development of acute bronchiolitis in the first two years of life. *An Pediatr (Engl Ed)*. Jun 2021;94(6):385-395. doi:10.1016/j.anpede.2020.05.011
9. Maitre L, Julvez J, Lopez-Vicente M, et al. Early-life environmental exposure determinants of child behavior in Europe: A longitudinal, population-based study. *Environ Int*. Aug 2021;153:106523. doi:10.1016/j.envint.2021.106523
10. Martin-Pujol A, Fernandez E, Schiaffino A, et al. Tobacco smoking, exposure to second-hand smoke, and asthma and wheezing in schoolchildren: a cross-sectional study. *Acta Paediatr*. Jul 2013;102(7):e305-9. doi:10.1111/apa.12232
11. Suarez Lopez de Vergara RG, Galvan Fernandez C, Oliva Hernandez C, Aguirre-Jaime A, Vazquez Moncholi C, Grupo de Trabajo sobre Tabaquismo de la Infancia y Adolescencia de la Sociedad Espanola de Neumologia P. [Environmental tobacco smoke exposure in children and its relationship with the severity of asthma]. *An Pediatr (Barc)*. Jan 2013;78(1):35-42. Exposicion al humo de tabaco del nino asmatico y su asociacion con la gravedad del asma. doi:10.1016/j.anpedi.2011.12.008
12. Padron A, Galan I, Rodriguez-Artalejo F. Second-hand smoke exposure and psychological distress in adolescents. A population-based study. *Tob Control*. Jul 2014;23(4):302-7. doi:10.1136/tobaccocontrol-2012-050548
13. Padron A, Galan I, Garcia-Esquinas E, Fernandez E, Ballbe M, Rodriguez-Artalejo F. Exposure to secondhand smoke in the home and mental health in children: a population-based study. *Tob Control*. May 2016;25(3):307-12. doi:10.1136/tobaccocontrol-2014-052077
14. Alicea-Alvarez N, Foppiano Palacios C, Ortiz M, Huang D, Reeves K. Path to health asthma study: A survey of pediatric asthma in an urban community. *J Asthma*. Apr 2017;54(3):273-278. doi:10.1080/02770903.2016.1216564

15. Arechavala T, Continente X, Perez-Rios M, et al. Second-hand smoke exposure in homes with children: assessment of airborne nicotine in the living room and children's bedroom. *Tob Control*. Jul 2018;27(4):399-406. doi:10.1136/tobaccocontrol-2017-053751
16. Lopez MJ, Arechavala T, Continente X, Schiaffino A, Perez-Rios M, Fernandez E. Social inequalities in secondhand smoke exposure in children in Spain. *Tob Induc Dis*. 2018;16(April):14. doi:10.18332/tid/85717
17. Arechavala T, Continente X, Perez-Rios M, Schiaffino A, Fernandez E, Lopez MJ. Sociodemographic factors associated with secondhand smoke exposure and smoking rules in homes with children. *Eur J Public Health*. Oct 1 2019;29(5):843-849. doi:10.1093/eurpub/ckz054
18. Continente X, Arechavala T, Fernandez E, et al. Burden of respiratory disease attributable to secondhand smoke exposure at home in children in Spain (2015). *Prev Med*. Jun 2019;123:34-40. doi:10.1016/j.ypmed.2019.02.028
19. Diez-Izquierdo A, Cassanello Penarroya P, Cartanya-Hueso A, et al. [Prevalence of smoke-free homes and passive exposure to tobacco in pediatric population (children from 3 to 36 months)]. *Rev Esp Salud Publica*. Jul 16 2019;93:1-13. Prevalencia de hogares libres de humo y exposicion pasiva al tabaco en poblacion pediatrica (ninos de 3 a 36 meses).
20. Lletjos P, Continente X, Arechavala T, et al. [Association between exposure to second-hand smoke and health status in children]. *Gac Sanit*. Jul-Aug 2020;34(4):363-369. Asociacion entre el humo ambiental de tabaco y el estado de salud en la poblacion infantil. doi:10.1016/j.gaceta.2018.10.006
21. Henderson E, Continente X, Fernandez E, et al. Secondhand smoke exposure and other signs of tobacco consumption at outdoor entrances of primary schools in 11 European countries. *Sci Total Environ*. Nov 15 2020;743:140743. doi:10.1016/j.scitotenv.2020.140743
22. Continente X, Rodriguez A, Perez-Rios M, Schiaffino A, Fernandez E, Lopez MJ. Factors related to caregivers' risk perception of secondhand smoke exposure on children's health. *Tob Induc Dis*. 2021;19(December):93. doi:10.18332/tid/143318
23. Gonzalez-Barcala FJ, Pertega S, Perez Castro T, et al. Exposure to paracetamol and asthma symptoms. *Eur J Public Health*. Aug 2013;23(4):706-10. doi:10.1093/eurpub/cks061
24. Román Almendros M, García-Campaña A, Hidalgo-Lacalle M, López-León M. Perfil de las gestantes y grado de exposición al humo del tabaco. *Matronas Prof*. 2018;19 135-141.
25. Hernandez-Martinez C, Arija Val V, Escribano Subias J, Canals Sans J. A longitudinal study on the effects of maternal smoking and secondhand smoke exposure during pregnancy on neonatal neurobehavior. *Early Hum Dev*. Jun 2012;88(6):403-8. doi:10.1016/j.earlhumdev.2011.10.004
26. McBride D, Keil T, Grabenhenrich L, et al. The EuroPrevall birth cohort study on food allergy: baseline characteristics of 12,000 newborns and their families from nine European countries. *Pediatr Allergy Immunol*. May 2012;23(3):230-9. doi:10.1111/j.1399-3038.2011.01254.x
27. Casas M, Valvi D, Luque N, et al. Dietary and sociodemographic determinants of bisphenol A urine concentrations in pregnant women and children. *Environ Int*. Jun 2013;56:10-8. doi:10.1016/j.envint.2013.02.014
28. Ribot B, Isern R, Hernandez-Martinez C, Canals J, Aranda N, Arija V. [Effects of tobacco habit, second-hand smoking and smoking cessation during pregnancy on newborn's health]. *Med Clin (Barc)*. Jul 22 2014;143(2):57-63. Impacto del tabaquismo,

la exposicion pasiva al tabaco y el dejar de fumar sobre la salud del recien nacido. doi:10.1016/j.medcli.2013.09.040
29. Hernandez-Martinez C, Voltas Moreso N, Ribot Serra B, Arija Val V, Escribano Macias J, Canals Sans J. Effects of Prenatal Nicotine Exposure on Infant Language Development: A Cohort Follow Up Study. *Matern Child Health J*. Apr 2017;21(4):734-744. doi:10.1007/s10995-016-2158-y
30. Roige-Castellvi J, Murphy M, Hernandez-Martinez C, et al. The effect of prenatal smoke exposure on child neuropsychological function: a prospective mother-child cohort study. *J Reprod Infant Psychol*. Feb 2020;38(1):25-37. doi:10.1080/02646838.2019.1580350
31. Iniguez C, Esplugues A, Sunyer J, et al. Prenatal Exposure to NO2 and Ultrasound Measures of Fetal Growth in the Spanish INMA Cohort. *Environ Health Perspect*. Feb 2016;124(2):235-42. doi:10.1289/ehp.1409423
32. Ruano-Ravina A, Garcia-Lavandeira JA, Torres-Duran M, et al. Leisure time activities related to carcinogen exposure and lung cancer risk in never smokers. A case-control study. *Environ Res*. Jul 2014;132:33-7. doi:10.1016/j.envres.2014.03.027
33. Torres-Duran M, Ruano-Ravina A, Parente-Lamelas I, et al. Lung cancer in never-smokers: a case-control study in a radon-prone area (Galicia, Spain). *Eur Respir J*. Oct 2014;44(4):994-1001. doi:10.1183/09031936.00017114
34. Almirall J, Serra-Prat M, Bolibar I, et al. Passive smoking at home is a risk factor for community-acquired pneumonia in older adults: a population-based case-control study. *BMJ Open*. Jun 13 2014;4(6):e005133. doi:10.1136/bmjopen-2014-005133
35. Torres-Duran M, Ruano-Ravina A, Parente-Lamelas I, et al. Alpha-1 Antitrypsin Deficiency and Lung Cancer Risk: A Case-Control Study in Never-Smokers. *J Thorac Oncol*. Sep 2015;10(9):1279-1284. doi:10.1097/JTO.0000000000000609
36. Torres-Duran M, Ruano-Ravina A, Kelsey KT, et al. Environmental tobacco smoke exposure and EGFR and ALK alterations in never smokers' lung cancer. Results from the LCRINS study. *Cancer Lett*. Dec 28 2017;411:130-135. doi:10.1016/j.canlet.2017.09.042
37. Gonzalez Romero MP, Cuevas-Fernandez FJ, Marcelino-Rodriguez I, et al. [Application of the Smoking Scale for Primary Care (ETAP) in clinical practice]. *Aten Primaria*. Aug-Sep 2018;50(7):414-421. Aplicacion de la Escala de Tabaquismo para Atencion Primaria (ETAP) en la practica clinica. doi:10.1016/j.aprim.2017.05.010
38. Molina-Montes E, Van Hoogstraten L, Gomez-Rubio P, et al. Pancreatic Cancer Risk in Relation to Lifetime Smoking Patterns, Tobacco Type, and Dose-Response Relationships. *Cancer Epidemiol Biomarkers Prev*. May 2020;29(5):1009-1018. doi:10.1158/1055-9965.EPI-19-1027
39. Torres-Duran M, Curiel-Garcia MT, Ruano-Ravina A, et al. Small-cell lung cancer in never-smokers. *ESMO Open*. Apr 2021;6(2):100059. doi:10.1016/j.esmoop.2021.100059
40. Torres-Duran M, Ruano-Ravina A, Parente-Lamelas I, et al. Residential radon and lung cancer characteristics in never smokers. *Int J Radiat Biol*. Aug 2015;91(8):605-10. doi:10.3109/09553002.2015.1047985
41. Sunyer J, Garcia-Esteban R, Castilla AM, et al. Exposure to second-hand smoke and reproductive outcomes depending on maternal asthma. *Eur Respir J*. Aug 2012;40(2):371-6. doi:10.1183/09031936.00091411
42. Larranaga I, Santa-Marina L, Begiristain H, et al. Socio-economic inequalities in health, habits and self-care during pregnancy in Spain. *Matern Child Health J*. Sep 2013;17(7):1315-24. doi:10.1007/s10995-012-1134-4

43. Ortega-Garcia JA, Perales JE, Carceles-Alvarez A, et al. Long term follow-up of a tobacco prevention and cessation program in cystic fibrosis patients. *Adicciones*. Mar 2 2016;28(2):99-107. Seguimiento a largo plazo de un programa de prevencion y cesacion tabaquica en pacientes con fibrosis quistica. doi:10.20882/adicciones.778
44. Lidon-Moyano C, Fu M, Ballbe M, et al. Impact of the Spanish smoking laws on tobacco consumption and secondhand smoke exposure: A longitudinal population study. *Addict Behav*. Dec 2017;75:30-35. doi:10.1016/j.addbeh.2017.06.016
45. Perez-de-Arcelus M, Toledo E, Martinez-Gonzalez MA, Martin-Calvo N, Fernandez-Montero A, Moreno-Montanes J. Smoking and incidence of glaucoma: The SUN Cohort. *Medicine (Baltimore)*. Jan 2017;96(1):e5761. doi:10.1097/MD.0000000000005761
46. Roman-Galvez RM, Amezcua-Prieto C, Olmedo-Requena R, Lewis-Mikhael Saad AM, Martinez-Galiano JM, Bueno-Cavanillas A. Partner smoking influences whether mothers quit smoking during pregnancy: a prospective cohort study. *BJOG*. Jun 2018;125(7):820-827. doi:10.1111/1471-0528.14986
47. Flexeder C, Zock JP, Jarvis D, et al. Second-hand smoke exposure in adulthood and lower respiratory health during 20 year follow up in the European Community Respiratory Health Survey. *Respir Res*. Feb 14 2019;20(1):33. doi:10.1186/s12931-019-0996-z
48. Olivieri M, Murgia N, Carsin AE, et al. Effects of smoking bans on passive smoking exposure at work and at home. The European Community respiratory health survey. *Indoor Air*. Jul 2019;29(4):670-679. doi:10.1111/ina.12556
49. Iniguez C, Ballester F, Amoros R, Murcia M, Plana A, Rebagliato M. Active and passive smoking during pregnancy and ultrasound measures of fetal growth in a cohort of pregnant women. *J Epidemiol Community Health*. Jun 2012;66(6):563-70. doi:10.1136/jech.2010.116756
50. Ruano-Ravina A, Cameselle-Lago C, Torres-Duran M, et al. Indoor Radon Exposure and COPD, Synergic Association? A Multicentric, Hospital-Based Case-Control Study in a Radon-Prone Area. *Arch Bronconeumol*. Oct 2021;57(10):630-636. doi:10.1016/j.arbr.2020.11.020
51. Villaverde Royo MV, Marin Izaguerri MP, Requeno Jarabo MN, Val Esco L, Coronas Mateos S, Cordoba-Garcia R. [Impact of the smoke-free legislation on the prevalence and referred time exposure to the environmental tobacco smoke in Zaragoza]. *Aten Primaria*. Oct 2012;44(10):603-10. Impacto de la regulacion de espacios sin humo en la exposicion referida al humo ambiental de tabaco en Zaragoza. doi:10.1016/j.aprim.2012.02.010
52. Clemente Jimenez ML, Bartolome Moreno C, Rubio Aranda E, et al. [Spanish opinions on tobacco smoke-free areas]. *Aten Primaria*. Mar 2012;44(3):138-44. Actitudes de los espanoles frente a los espacios libres de humo de tabaco. doi:10.1016/j.aprim.2011.01.015
53. Martínez-Sánchez JM, Fu M, Schiaffino A, et al. Exposición al humo ambiental del tabaco en el hogar y el tiempo libre según el día de la semana (laborable y no laborable) en Barcelona. *Adicciones*. 2012;24(2):173-178. doi:10.20882/adicciones.110
54. Ortega-Garcia JA, Gutierrez-Churango JE, Sanchez-Sauco MF, et al. Head circumference at birth and exposure to tobacco, alcohol and illegal drugs during early pregnancy. *Childs Nerv Syst*. Mar 2012;28(3):433-9. doi:10.1007/s00381-011-1607-6
55. Jimenez-Muro A, Samper MP, Marqueta A, Rodriguez G, Nerin I. [Prevalence of smoking and second-hand smoke exposure: differences between Spanish and immigrant pregnant women]. *Gac Sanit*. Mar-Apr 2012;26(2):138-44. Prevalencia de

tabaquismo y exposicion al humo ambiental de tabaco en las mujeres embarazadas: diferencias entre espanolas e inmigrantes. doi:10.1016/j.gaceta.2011.07.015
56. Aurrekoetxea JJ, Murcia M, Rebagliato M, et al. Determinants of self-reported smoking and misclassification during pregnancy, and analysis of optimal cut-off points for urinary cotinine: a cross-sectional study. *BMJ Open*. Jan 24 2013;3(1)doi:10.1136/bmjopen-2012-002034
57. Mateos-Vilchez PM, Aranda-Regules JM, Diaz-Alonso G, et al. [Smoking prevalence and associated factors during pregnancy in Andalucia 2007-2012]. *Rev Esp Salud Publica*. May-Jun 2014;88(3):369-81. Prevalencia de tabaquismo durante el embarazo y factores asociados en Andalucia 2007-2012 (*). doi:10.4321/S1135-57272014000300007
58. Sureda X, Martinez-Sanchez JM, Fu M, et al. Impact of the Spanish smoke-free legislation on adult, non-smoker exposure to secondhand smoke: cross-sectional surveys before (2004) and after (2012) legislation. *PLoS One*. 2014;9(2):e89430. doi:10.1371/journal.pone.0089430
59. Aurrekoetxea JJ, Murcia M, Rebagliato M, et al. Factors associated with second-hand smoke exposure in non-smoking pregnant women in Spain: self-reported exposure and urinary cotinine levels. *Sci Total Environ*. Feb 1 2014;470-471:1189-96. doi:10.1016/j.scitotenv.2013.10.110
60. Perez-Rios M, Santiago-Perez MI, Malvar A, et al. [Impact of the Spanish smoking laws on the exposure to environmental tobacco smoke in Galicia (2005-2011)]. *Gac Sanit*. Jan-Feb 2014;28(1):20-4. Impacto de las leyes de control del tabaquismo en la exposicion al humo ambiental de tabaco en Galicia (2005-2011). doi:10.1016/j.gaceta.2013.04.010
61. Galan I, Mayo E, Lopez MJ, et al. Validity of self-reported exposure to second-hand smoke in hospitality venues. *Environ Res*. Aug 2014;133:1-3. doi:10.1016/j.envres.2014.04.029
62. Sureda X, Fernandez E, Martinez-Sanchez JM, et al. Secondhand smoke in outdoor settings: smokers' consumption, non-smokers' perceptions, and attitudes towards smoke-free legislation in Spain. *BMJ Open*. Apr 8 2015;5(4):e007554. doi:10.1136/bmjopen-2014-007554
63. Ballbe M, Sureda X, Martinez-Sanchez JM, et al. Secondhand smoke in psychiatric units: patient and staff misperceptions. *Tob Control*. Oct 2015;24(e3):e212-20. doi:10.1136/tobaccocontrol-2014-051585
64. Ballbe M, Martinez-Sanchez JM, Gual A, et al. Association of second-hand smoke exposure at home with psychological distress in the Spanish adult population. *Addict Behav*. Nov 2015;50:84-8. doi:10.1016/j.addbeh.2015.06.020
65. Fernandez E, Fu M, Perez-Rios M, Schiaffino A, Sureda X, Lopez MJ. Changes in Secondhand Smoke Exposure After Smoke-Free Legislation (Spain, 2006-2011). *Nicotine Tob Res*. Nov 1 2017;19(11):1390-1394. doi:10.1093/ntr/ntx040
66. Martinez C, Mendez C, Sanchez M, Martinez-Sanchez JM. Attitudes of students of a health sciences university towards the extension of smoke-free policies at the university campuses of Barcelona (Spain). *Gac Sanit*. Mar-Apr 2017;31(2):132-138. doi:10.1016/j.gaceta.2016.08.009
67. Vinolas N, Garrido P, Isla D, et al. Lung Cancer in Never-Smoking Women: A Sub-Analysis of the Spanish Female-Specific Database WORLD07. *Cancer Invest*. May 28 2017;35(5):358-365. doi:10.1080/07357907.2017.1295461
68. Martinez-Sanchez JM, Gonzalez-Marron A, Martin-Sanchez JC, et al. Validity of self-reported intensity of exposure to second-hand smoke at home against

environmental and personal markers. *Gac Sanit*. Jul-Aug 2018;32(4):393-395. doi:10.1016/j.gaceta.2017.08.002
69. Sureda X, Bilal U, Fernandez E, et al. Second-hand smoke exposure in outdoor hospitality venues: Smoking visibility and assessment of airborne markers. *Environ Res*. Aug 2018;165:220-227. doi:10.1016/j.envres.2018.04.024
70. Fu M, Fernandez E, Martinez-Sanchez JM, et al. Second-hand smoke exposure in indoor and outdoor areas of cafes and restaurants: Need for extending smoking regulation outdoors? *Environ Res*. Jul 2016;148:421-428. doi:10.1016/j.envres.2016.04.024
71. Miguez MC, Pereira B. [Effects of active and/or passive smoking during pregnancy and the postpartum period]. *An Pediatr (Engl Ed)*. Oct 8 2020;95(4):222-232. Repercusiones del consumo de tabaco activo y/o pasivo en el embarazo y postparto. doi:10.1016/j.anpedi.2020.07.029
72. Lidon-Moyano C, Fu M, Perez-Ortuno R, et al. Third-hand exposure at homes: Assessment using salivary cotinine. *Environ Res*. May 2021;196:110393. doi:10.1016/j.envres.2020.110393
73. Rebollar Álvarez A, Justo Gil S, Rey Brandariz J, González Carreño C, Gómez-Chacón Galán C. Tobacco use in Spain during COVID-19 lockdown: an evaluation through social media. *Rev Esp Salud Publica*. 2021;1(2)
74. Nogueira SO, Fu M, Lugo A, et al. Non-smokers' and smokers' support for smoke-free legislation in 14 indoor and outdoor settings across 12 European countries. *Environ Res*. Mar 2022;204(Pt C):112224. doi:10.1016/j.envres.2021.112224
75. Tack SHSPI; Henderson E, Lugo A, et al. Secondhand smoke presence in outdoor areas in 12 European countries. *Environ Res*. Apr 2021;195:110806. doi:10.1016/j.envres.2021.110806

© 2024 Blanco-Ferreiro A. et al.
